# CoSIA: an R Bioconductor package for CrOss Species Investigation and Analysis

**DOI:** 10.1093/bioinformatics/btad759

**Published:** 2023-12-18

**Authors:** Anisha Haldar, Vishal H Oza, Nathaniel S DeVoss, Amanda D Clark, Brittany N Lasseigne

**Affiliations:** The Department of Cell, Developmental and Integrative Biology, Heersink School of Medicine, The University of Alabama at Birmingham, Birmingham, AL 35294, United States; The Department of Cell, Developmental and Integrative Biology, Heersink School of Medicine, The University of Alabama at Birmingham, Birmingham, AL 35294, United States; The Department of Cell, Developmental and Integrative Biology, Heersink School of Medicine, The University of Alabama at Birmingham, Birmingham, AL 35294, United States; The Department of Cell, Developmental and Integrative Biology, Heersink School of Medicine, The University of Alabama at Birmingham, Birmingham, AL 35294, United States; The Department of Cell, Developmental and Integrative Biology, Heersink School of Medicine, The University of Alabama at Birmingham, Birmingham, AL 35294, United States

## Abstract

**Summary:**

High-throughput sequencing technologies have enabled cross-species comparative transcriptomic studies; however, there are numerous challenges for these studies due to biological and technical factors. We developed CoSIA (Cross-Species Investigation and Analysis), a Bioconductor R package and Shiny app that provides an alternative framework for cross-species transcriptomic comparison of non-diseased wild-type RNA sequencing gene expression data from Bgee across tissues and species (human, mouse, rat, zebrafish, fly, and nematode) through visualization of variability, diversity, and specificity metrics.

**Availability and implementation:**

https://github.com/lasseignelab/CoSIA.

## 1 Introduction

With the advent of high-throughput sequencing technologies (Goodwin *et al.* 2016), there has been an explosion in the generation of gene expression data across multiple species (Mutz *et al.* 2013). Providing an excellent opportunity to leverage this available data to better study human and model organism gene expression patterns in a biomedical context, cross-species comparative studies have elucidated disease mechanisms, evolutionary patterns, and developmental differences (LoVerso and Cui 2015). However, cross-species gene expression comparison is challenging due to biological and technical factors affecting the measurements (Conesa *et al.* 2016, Chung *et al.* 2021). Previous studies have implemented a variety of comparison methods (Zhu *et al.* 2014, Sudmant *et al.* 2015, Söllner *et al.* 2017, Panahi *et al.* 2019, Wang *et al.* 2020, Bastian *et al.* 2021, García de la Torre *et al.* 2021, Liu *et al.* 2023) that involve either taking into account the evolutionary relationships between the species or rigorous statistical assumptions to account for species-level differences in gene expression (Fisher 1948, Stuart *et al.* 2003, Hu *et al.* 2006, Lu *et al.* 2006, 2007, 2010, Campain and Yang, 2010, Le *et al.* 2010, Tseng *et al.* 2012, Kristiansson *et al.* 2013). We developed CoSIA (Cross-Species Investigation and Analysis), an R package and associated Shiny app, which provides an alternative framework for cross-species RNA expression visualization and comparison across tissues and species using variability metrics. CoSIA allows users to calculate and visualize variability, diversity, and specificity metrics across Homo sapiens and five species commonly used in biomedical research: *Mus musculus*, *Rattus norvegicus*, *Danio rerio*, *Drosophila melanogaster*, and *Caenorhabditis elegans*.

## 2 Implementation

**Figure 1.**
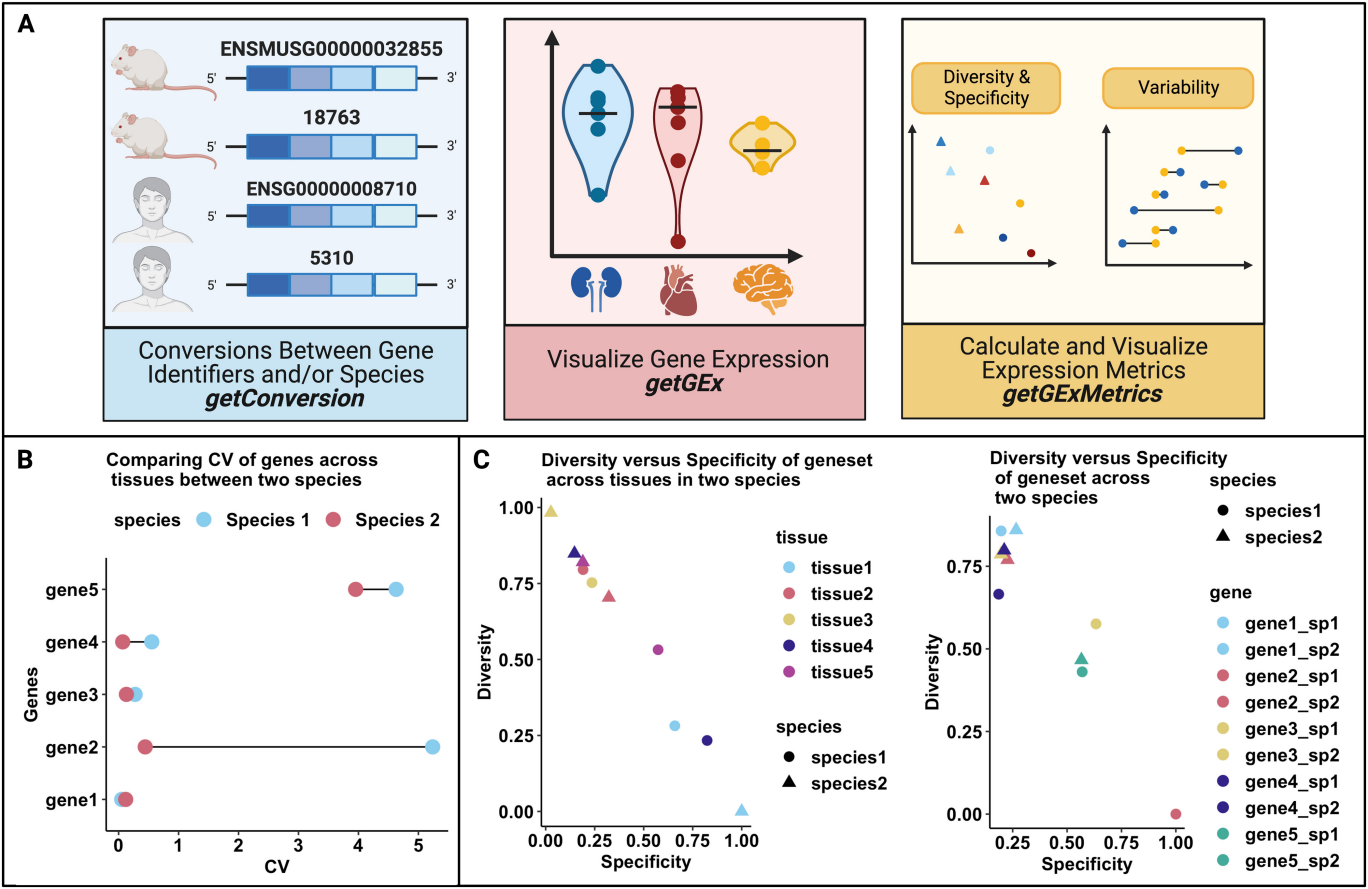
(A) The CoSIA R package workflow. (B) The coefficient of variation cross-species comparison analysis depicts a lollipop plot of example data used to show median-based CV gene comparison across species. This example highlights genes with high variability (gene5), low variability (gene4, gene3, and gene1), and species-specific variability (gene2) in expression across tissues. (C) The diversity and specificity cross-species comparison analysis depicts scatter plots of example data (Supplementary Table S2) showing diversity and specificity metric for tissues and genes. This example highlights the differences in diversity and specificity across tissues (tissue1 and tissue3 in species2), and the similarities in diversity and specificity across species (gene1, gene2, gene3, and gene4 in species2). Figure was created with BioRender.com

CoSIA (Haldar *et al.* 2023), is a Bioconductor package accessible as part of the Bioconductor 3.17 release. CoSIA allows for relative cross-species comparison of non-diseased wild-type RNA sequencing gene expression data across tissues and species. Specifically, CoSIA implements methods for mapping gene identifiers and orthologs, visualizing gene expression by tissue and species, and comparing cross-species gene expression metrics, as shown in Fig. 1A.

### 2.1 Mapping between gene identifiers and orthologs (getConversion)

CoSIA provides the user with a streamlined method for converting between different gene identifiers and cross-species ortholog mapping. The current gene identifiers supported are Ensembl IDs, Entrez IDs, and Gene Symbols. These conversions are performed with the BiomaRt (Durinck *et al.* 2005) and AnnotationDBI (Pagès *et al.* 2022) packages, while ortholog gene mapping is performed using the NCBI Homologene Database (Sayers *et al.* 2022) and the NCBI Eukaryotic Genome Annotation Pipeline Database (Thibaud-Nissen *et al.* 2016).

### 2.2 Visualizing gene expression data (getGEx)

CoSIA uses the Bioconductor Data Package CoSIAdata (EH7858, EH7859, EH7860, EH7861, EH7862, EH7863) hosted on ExperimentHub, which contains variance stabilized transcript per million (TPM) gene expression values of non-diseased wild-type RNA-seq read counts we retrieved using BgeeDB [package v2.26.0; database v15.0] (Komljenovic *et al.* 2016). The variance stabilization is done using the Variance Stabilizing Transformation (VST) method implemented in the DESeq2 (Love *et al.* 2014) package. These values are parsed (depending on the user’s choice of genes, tissues, and species) into a data frame that can be visualized as an interactive violin plot through the plotSpeciesGEx and plotTissueGEx plotting functions. More information about data preprocessing can be found in Supplementary Methods.

### 2.3 Calculating and visualizing gene expression metric data (getGExMetrics)

Comparing transcriptional profiles across species has been challenging because of differences in gene expression patterns and batch effects. Previous attempts at directly comparing expression between species using various normalization techniques (Dunn *et al.* 2018) have been shown to be affected by annotation depth and quality (Oziolor *et al.* 2021). Other studies (Breschi *et al.* 2016) have shown that each gene has a specific pattern, with some genes showing higher variation between organs within the same species compared to variation between species and vice versa. Direct comparison methods do not account for these aspects. To overcome these challenges, we implemented variability (Coefficient of Variation), diversity, and specificity (Shannon entropy-based) metrics that allow for relative comparison of gene expression patterns between species. To understand how these metrics work, we have simulated the expression data of five genes and its orthologs across two species and five tissues (Supplementary Table S2). The metrics calculated on these genes are plotted in Fig. 1B and C.

#### 2.3.1 Coefficeint of variation

The coefficeint of variation (CV) in CoSIA is calculated as the standard deviation over the median using VST values. CoSIA provides two approaches for calculating the CV. ‘CV Tissue’ calculates the CV of a set of user-supplied genes across the specified tissues, while ‘CV Species’ calculates the CV of user-supplied genes across the specified species. The calculated CVs are returned as a data frame and visualized as lollipop plots using the plotCVGEx plotting function.s shown in Fig. 1B, Gene 2 has high variation in expression across tissues in species 1 but not in species 2. However, Genes 1 and 3 have no variation in expression across both species, thus their expression does not change much across tissues in both species.

#### 2.3.2 Diversity and specificity

To calculate the diversity and specificity metrics, we first calculate the median of the variance stabilized TPM values for each gene in a specific tissue in a given species. These median values are rescaled using min-max scaling, which preserves the distribution of values but rescales the values between 0 and 1. These values are used to calculate the diversity and specificity metrics as described in (Martínez and Reyes-Valdés 2008). Briefly, diversity and specificity in the context of gene expression across tissues are quantified using Shannon entropy. Diversity refers to the degree of heterogeneity or variability in the expression patterns of a gene across different tissues. It captures the distribution and relative frequency of expression across tissues, with higher diversity indicating a more evenly distributed expression pattern. Specificity, on the other hand, measures the level of concentration or selectivity of gene expression within a particular tissue. It assesses the extent to which a gene’s expression is confined to a specific tissue, with higher specificity indicating a more restricted or specialized expression pattern within that tissue. The range of diversity and specificity is between 0 and 1, with 0 being low and 1 being high. Another important thing to note is that they are inversely related (Jones *et al.* 2023). Thus, a highly diverse gene will have similar expression across tissues but low specificity. A highly specific gene will have higher expression in one tissue compared to other tissues. CoSIA allows for four different calculations of diversity and specificity in a species: (i) ‘DS Gene’ compares user-specified genes across user-specified tissues, (ii) ‘DS Gene all’ compares user-specified genes across all tissues in a species, (iii) ‘DS Tissue’ compares user-specified tissues across user-specified genes, and (iv) ‘DS Tissue all’ compares user-specified tissues across all genes in a species. The metrics can either be exported as a data frame or visualized using diversity/specificity scatter plots using the plotDSGEx plotting function. The first half of Fig. 1C shows that the geneset expression is very specific in tissue 1 in species 2 compared to species 1. On the contrary, the geneset expression is very diverse in tissue 3 in species 2 compared to species 1. In the second half of Fig. 1C, we look at individual genes, where gene 2 has very specific expression in species 1; however, it is very diverse in species 2.

### 2.4 Shiny app

We have also implemented the CoSIA package as a Shiny app (DeVoss *et al.* 2023) hosted at (https://lasseignelab.shinyapps.io/CoSIA/) which provides a graphical user interface with similar functionalities as the package.

## 3 Conclusion

Direct comparison of gene expression between species is complex as it is confounded by differing levels of expression and function of orthologous genes across species. Here, we provide the CoSIA package and Shiny app to facilitate the relative comparison of gene expression by summary metrics (i.e., coefficient of variation, diversity, and specificity) in six species. By leveraging the Bgee database of species-specific RNA-Seq expression data, we provide tools for the robust comparison of gene expression values across both species and tissues. An important caveat is these metrics are useful for visualization of the variation in gene expression across tissues and species but should not be used for downstream analysis. We believe CoSIA will also aid biomedical researchers in selecting optimal model organisms for a given gene in a tissue of interest.

## Supplementary Material

btad759_Supplementary_Data

## Data Availability

The data underlying this article are available in Bioconductor data package CoSIAdata, at 10.18129/B9.bioc.CoSIAdata.

## References

[btad759-B1] Bastian FB , Roux J, Niknejad A et al The bgee suite: integrated curated expression atlas and comparative transcriptomics in animals. Nucleic Acids Res 2021;49:D831–47. 33037820 10.1093/nar/gkaa793 PMC7778977

[btad759-B2] Breschi A , Djebali S, Gillis J et al Gene-specific patterns of expression variation across organs and species. Genome Biol 2016;17:151. 27391956 10.1186/s13059-016-1008-y PMC4937605

[btad759-B3] Campain A , Yang YH. Comparison study of microarray meta-analysis methods. BMC Bioinformatics 2010;11:408. 20678237 10.1186/1471-2105-11-408 PMC2922198

[btad759-B4] Chung M , Bruno VM, Rasko DA et al Best practices on the differential expression analysis of multi-species RNA-seq. Genome Biol 2021;22:121. 33926528 10.1186/s13059-021-02337-8 PMC8082843

[btad759-B5] Conesa A , Madrigal P, Tarazona S et al A survey of best practices for RNA-seq data analysis. Genome Biol 2016;17:13. 26813401 10.1186/s13059-016-0881-8 PMC4728800

[btad759-B6] DeVoss NS , Clark A, Oza V et al CoSIA: cross-species investigation and analysis—ShinyApp. Apr 2023. https://lasseignelab.shinyapps.io/CoSIA/. 10.1093/bioinformatics/btad759 PMC10749757 38109675

[btad759-B7] Dunn CW , Zapata F, Munro C et al Pairwise comparisons across species are problematic when analyzing functional genomic data. Proc Natl Acad Sci USA 2018;115:E409–17. 29301966 10.1073/pnas.1707515115 PMC5776959

[btad759-B8] Durinck S , Moreau Y, Kasprzyk A et al BioMart and bioconductor: a powerful link between biological databases and microarray data analysis. Bioinformatics 2005;21:3439–40. 16082012 10.1093/bioinformatics/bti525

[btad759-B9] Fisher RA. 224a: answer to question 14 on combining independent tests of significance. Am Stat 1948.

[btad759-B10] García de la Torre VS , Majorel-Loulergue C, Rigaill GJ et al Wide cross-species RNA-Seq comparison reveals convergent molecular mechanisms involved in nickel hyperaccumulation across dicotyledons. New Phytol 2021;229:994–1006. 32583438 10.1111/nph.16775

[btad759-B11] Goodwin S , McPherson JD, McCombie WR. Coming of age: ten years of next-generation sequencing technologies. Nat Rev Genet 2016;17:333–51. 27184599 10.1038/nrg.2016.49 PMC10373632

[btad759-B12] Haldar A , Oza VH, Clark AD et al *CoSIA: An Investigation Across Different Species and Tissues*. R package version 1.2.0. 2023. 10.18129/B9.bioc.CoSIA.

[btad759-B13] Hu P , Greenwood CMT, Beyene J. Statistical methods for meta-analysis of microarray data: a comparative study. Inf Syst Front 2006;8:9–20.

[btad759-B14] Jones EF , Haldar A, Oza VH et al Quantifying transcriptome diversity: a review. Brief Funct Genomics 2023. 10.1093/bfgp/elad019 PMC11484519 37225889

[btad759-B15] Komljenovic A , Roux J, Wollbrett J et al BgeeDB, an R package for retrieval of curated expression datasets and for gene list expression localization enrichment tests. F1000Res 2016;5:2748. 30467516 10.12688/f1000research.9973.1 PMC6113886

[btad759-B16] Kristiansson E , Österlund T, Gunnarsson L et al A novel method for cross-species gene expression analysis. BMC Bioinformatics 2013;14:70. 23444967 10.1186/1471-2105-14-70 PMC3679856

[btad759-B17] Le H-S , Oltvai ZN, Bar-Joseph Z. Cross-species queries of large gene expression databases. Bioinformatics 2010;26:2416–23. 20702396 10.1093/bioinformatics/btq451 PMC2944203

[btad759-B18] Liu J , Zhang Y, Zheng Y et al PlantExp: a platform for exploration of gene expression and alternative splicing based on public plant RNA-seq samples. Nucleic Acids Res 2023;51:D1483–91. 36271793 10.1093/nar/gkac917 PMC9825497

[btad759-B19] Love MI , Huber W, Anders S. Moderated estimation of fold change and dispersion for RNA-seq data with DESeq2. Genome Biol 2014;15:550. 25516281 10.1186/s13059-014-0550-8 PMC4302049

[btad759-B20] LoVerso PR , Cui F. A computational pipeline for Cross-Species analysis of RNA-seq data using R and bioconductor. Bioinform Biol Insights 2015;9:165–74. 26692761 10.4137/BBI.S30884 PMC4668955

[btad759-B21] Lu Y , Rosenfeld R, Bar-Joseph Z. Identifying cycling genes by combining sequence homology and expression data. Bioinformatics 2006;22:e314–22. 16873488 10.1093/bioinformatics/btl229

[btad759-B22] Lu Y , Mahony S, Benos PV et al Combined analysis reveals a core set of cycling genes. Genome Biol 2007;8:R146. 17650318 10.1186/gb-2007-8-7-r146 PMC2323241

[btad759-B23] Lu Y , Rosenfeld R, Nau GJ et al Cross species expression analysis of innate immune response. J Comput Biol 2010;17:253–68. 20377444 10.1089/cmb.2009.0147 PMC3198893

[btad759-B24] Martínez O , Reyes-Valdés MH. Defining diversity, specialization, and gene specificity in transcriptomes through information theory. Proc Natl Acad Sci USA 2008;105:9709–14. 18606989 10.1073/pnas.0803479105 PMC2443819

[btad759-B25] Mutz K-O , Heilkenbrinker A, Lönne M et al Transcriptome analysis using next-generation sequencing. Curr Opin Biotechnol 2013;24:22–30. 23020966 10.1016/j.copbio.2012.09.004

[btad759-B26] Oziolor E , Arat S, Martin M. Annotation depth confounds direct comparison of gene expression across species. BMC Bioinformatics 2021;22:499. 34654362 10.1186/s12859-021-04414-y PMC8518172

[btad759-B27] Pagès H , Carlson M, Falcon S et al AnnotationDbi: manipulation of SQLite-based annotations in bioconductor. R package version 1.58.0. 2022.

[btad759-B28] Panahi B , Frahadian M, Dums JT et al Integration of cross species RNA-seq Meta-Analysis and Machine-Learning models identifies the most important salt Stress-Responsive pathways in microalga dunaliella. Front Genet 2019;10:752. 31555319 10.3389/fgene.2019.00752 PMC6727038

[btad759-B29] Sayers EW , Bolton EE, Brister JR et al Database resources of the national center for biotechnology information. Nucleic Acids Res 2022;50:D20–6. 34850941 10.1093/nar/gkab1112 PMC8728269

[btad759-B30] Söllner JF , Leparc G, Hildebrandt T et al An RNA-Seq atlas of gene expression in mouse and rat normal tissues. Sci Data 2017;4:170185. 29231921 10.1038/sdata.2017.185 PMC5726313

[btad759-B31] Stuart JM , Segal E, Koller D et al A gene-coexpression network for global discovery of conserved genetic modules. Science 2003;302:249–55. 12934013 10.1126/science.1087447

[btad759-B32] Sudmant PH , Alexis MS, Burge CB. Meta-analysis of RNA-seq expression data across species, tissues and studies. Genome Biol 2015;16:287. 26694591 10.1186/s13059-015-0853-4 PMC4699362

[btad759-B33] Thibaud-Nissen F , DiCuccio M, Hlavina W et al P8008 the NCBI eukaryotic genome annotation pipeline. J Anim Sci 2016;94:184.

[btad759-B34] Tseng GC , Ghosh D, Feingold E. Comprehensive literature review and statistical considerations for microarray meta-analysis. Nucleic Acids Res 2012;40:3785–99. 22262733 10.1093/nar/gkr1265 PMC3351145

[btad759-B35] Wang N , Niger C, Li N et al Cross-Species RNA-Seq study comparing transcriptomes of enriched osteocyte populations in the tibia and skull. Front Endocrinol (Lausanne) 2020;11:581002. 33071985 10.3389/fendo.2020.581002 PMC7543096

[btad759-B36] Zhu Y , Li M, Sousa AMM et al XSAnno: a framework for building ortholog models in cross-species transcriptome comparisons. BMC Genomics 2014;15:343. 24884593 10.1186/1471-2164-15-343 PMC4035071

