# Supplementary material for: CoSIA: an R Bioconductor package for CrOss Species Investigation and Analysis
Source: Bioinformatics. 2023 Dec 18;39(12):btad759. doi: 10.1093/bioinformatics/btad759 (PMC10749757; doi:10.1093/bioinformatics/btad759)
Supplement: btad759_Supplementary_Data [file btad759_supplementary_data.docx]

**Table 1.** Package Dependencies for CoSIA. This table includes the package name and minimum version of the package required for use in CoSIA. Entries are sorted alphabetically.

| **Package Names** | **Minimum Version Required for CoSIA** |
| --- | --- |
| AnnotationDbi | 1.52.0 |
| annotationTools | 1.64.0 |
| BgeeDB | 2.26.0 |
| BiocStyle | 2.22.0 |
| biomaRt | 2.46.3 |
| dplyr | 1.0.7 |
| ExperimentHub | 2.7.0 |
| ggplot2 | 3.3.5 |
| homologene | 1.4.68.19 |
| knitr | 1.42 |
| magrittr | 2.0.1 |
| methods | 4.1.2 |
| org.Ce.eg.db | 3.12.0 |
| org.Dm.eg.db | 3.12.0 |
| org.Dr.eg.db | 3.12.0 |
| org.Hs.eg.db | 3.12.0 |
| org.Mm.eg.db | 3.12.0 |
| org.Rn.eg.db | 3.12.0 |
| plotly | 4.10.0 |
| qpdf | 1.3.0 |
| RColorBrewer | 1.1-2 |
| readr | 2.1.1 |
| rmarkdown | 2.20 |
| stats | 4.1.2 |
| stringr | 1.4.0 |
| testthat | 3.1.6 |
| tibble | 3.1.7 |
| tidyr | 1.2.0 |
| tidyselect | 1.1.2 |
| tidyverse | 1.3.1 |

**Table 2.** Median Variance Stabilized Transformation of RNA-seq Read Counts example data used to render Figure 1b and 1c

|  | tissue1 | tissue2 | tissue3 | tissue4 | tissue5 | species |
| --- | --- | --- | --- | --- | --- | --- |
| gene1_sp1 | 8.34 | 8.55 | 8.65 | 7.56 | 8.55 | species1 |
| gene2_sp1 | 1.6 | 1.6 | 1.6 | 1.6 | 20.34 | species1 |
| gene3_sp1 | 5.43 | 6.34 | 5.86 | 9.4 | 5.83 | species1 |
| gene4_sp1 | 6.34 | 12.35 | 12.35 | 6.32 | 5.32 | species1 |
| gene5_sp1 | 2.3 | 21.34 | 22.12 | 2.3 | 2.3 | species1 |
| gene1_sp2 | 4.54 | 3.45 | 4.67 | 4.78 | 4.66 | species2 |
| gene2_sp2 | 4.5 | 6.7 | 12.4 | 12.7 | 18.43 | species2 |
| gene3_sp2 | 1.3 | 1.4 | 1.6 | 1.8 | 1.7 | species2 |
| gene4_sp2 | 7.3 | 8.4 | 8.6 | 8.4 | 7.6 | species2 |
| gene5_sp2 | 1.8 | 18.2 | 17.4 | 1.8 | 2.2 | species2 |

**Figure S1.**

**A)** **B)**


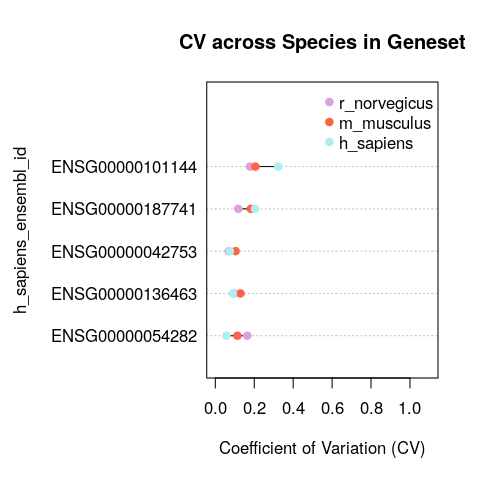

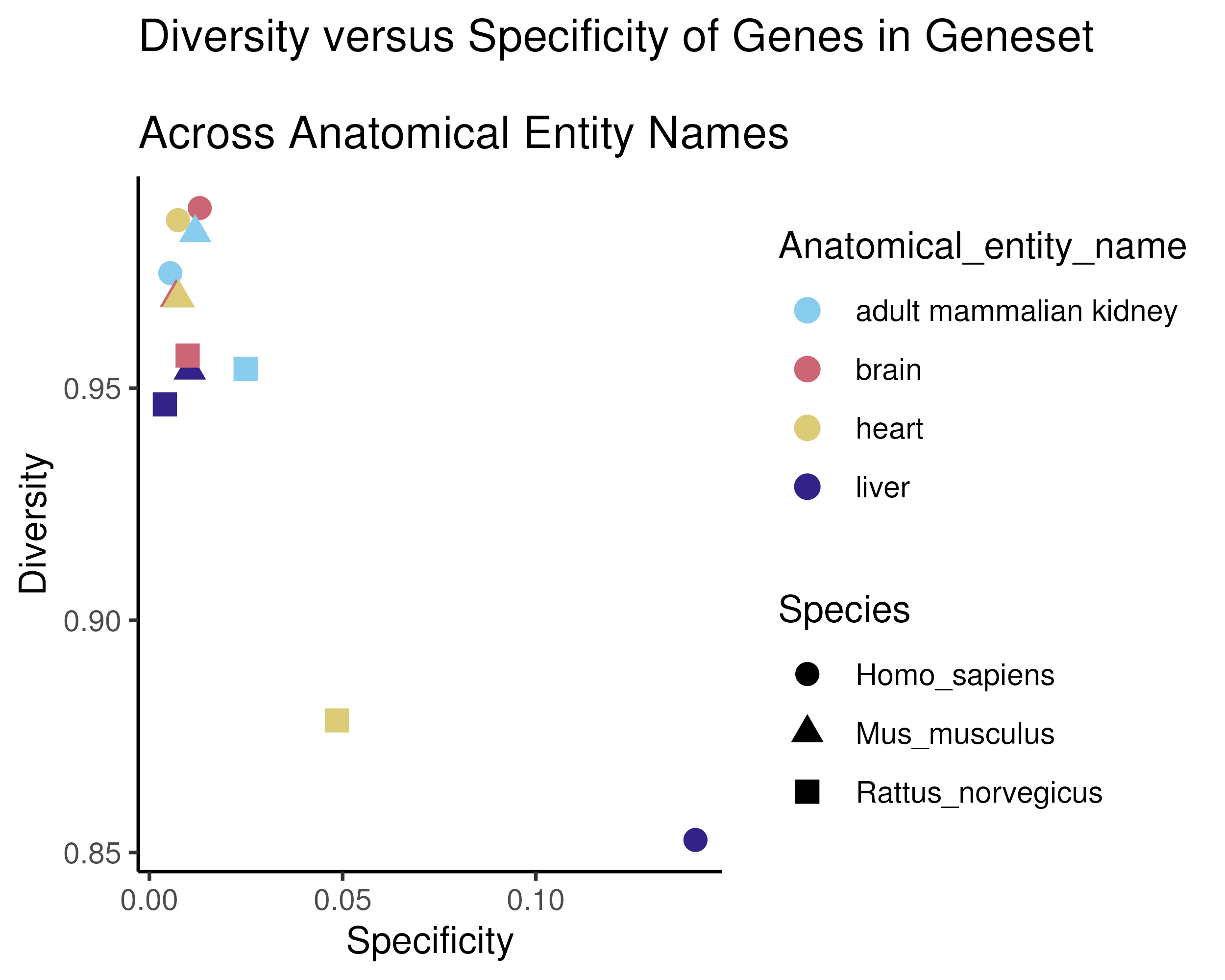


CoSIA metrics calculated on genes associated with monogenic kidney diseases in humans and its orthologs in mice and rats **(A)** The coefficient of variation cross-species comparison for **(B)** Diversity and specificity metrics across kidney, brain, heart, and liver.

### Methods:

Data preprocessing

For each experiment/library reported in Bgee for the six model organisms, we applied the vst function (using blind dispersion) of the DESeq2 package to obtain the vst read counts for each gene by experiment. These values are used for visualization of the gene expression. To calculate the CV, diversity, and specificity metrics, first we took these vst read counts and calculated MAD (Median Absolute Deviation) for each gene across experiments. We then filtered and kept only those genes whose MAD value was less than 1 across experiments, thus ensuring genes have consistent expression across experiments. For the filtered genes, we took the vst values for each experiment, scaled it using min-max normalization, and calculated the median value for a given gene across experiments in each tissue in a given organism. We then used min-max scaling so that values are between 0 and 1, while preserving the distribution and allowing for accurate calculation of metrics. Thus, we had a scaled median vst read count for each gene in each tissue in a given organism, which is used to calculate the metrics.
